# A Case of Electrotherapy

**Published:** 1872-01

**Authors:** 


A Case of Electrotherapy.—J. W. Holland, M.D., (American Practitioner, January, 1872,) reports a case of "nervous deafness" attributed to an attack of remittent fever fifteen years previously. A careful examination showed no lesion of structure in parts accessible to inspection, nor were there any indications of organic mischief. "The feeblest current from a volta faradaic coil that was perceptible to the hand was employed upon both ears successively. The auditory canal was filled with water, and the positive electrode, in the shape of a metallic conductor, insulated—save at the end, where it terminated in a silver ball about the size of a barleycorn—was inserted nearly to the tympanum. The circuit was completed by the negative sponge electrode being placed upon the superior cervical ganglion of the same or opposite side. A sitting of five minute's length resulted in the immediate and considerable improvement of both ears. On reaching the street, the hearing was so much more acute than when the patient entered the office as to impel him to remark that 'it was the revelation of a new world.' Where before the tick of an uucommonly noisy watch could not be heard even when pressed hard upon the ear and mastoid process, the renewed sensibility enabled him to hear it nearly an inch away. The revival of the auditory nerves gained fresh force from each electrical sitting; and one may fairly presume that, as five applications have accomplished so much, by continued excitation the torpor may eventually be entirely removed."

